# The International Conference on Intelligent Biology and Medicine (ICIBM) 2019: bioinformatics methods and applications for human diseases

**DOI:** 10.1186/s12859-019-3240-4

**Published:** 2019-12-20

**Authors:** Zhongming Zhao, Yulin Dai, Chi Zhang, Ewy Mathé, Lai Wei, Kai Wang

**Affiliations:** 1 Center for Precision Health, School of Biomedical Informatics, The University of Texas Health Science Center at Houston, Houston, TX 77030 USA; 2 Department of Medical and Molecular Genetics, Indiana University School of Medicine, Indianapolis, IN 46202 USA; 3 Department of Biomedical Informatics, The Ohio State University College of Medicine, Columbus, OH 43214 USA; 4 Raymond G. Perelman Center for Cellular and Molecular Therapeutics, Children’s Hospital of Philadelphia, Philadelphia, PA 19104 USA; 5 Department of Pathology and Laboratory Medicine, University of Pennsylvania, Philadelphia, PA 19104 USA

## Abstract

Between June 9–11, 2019, the International Conference on Intelligent Biology and Medicine (ICIBM 2019) was held in Columbus, Ohio, USA. The conference included 12 scientific sessions, five tutorials or workshops, one poster session, four keynote talks and four eminent scholar talks that covered a wide range of topics in bioinformatics, medical informatics, systems biology and intelligent computing. Here, we describe 13 high quality research articles selected for publishing in BMC Bioinformatics.

## Introduction

The 2019 International Conference on Intelligent Biology and Medicine (ICIBM 2019) provided a multidisciplinary forum for computational scientists and experimental biologists to share their most recent findings in the field of cancer genomics, systems biology, medical informatics, big data analytics and machine learning, among others. The conference was held on June 9–11, 2019, in Columbus, Ohio, USA. Approximately 200 researchers and students across the world attended the meeting. In this special issue, we have collected 13 original research articles reflecting the cutting-edge researches in bioinformatics. As the advance of all kinds of omics studies, bioinformatics has become the indispensable powerhouse behinds all analyses. This is reflected in our selection, as these papers cover traditional areas in genomics, transcriptomics, proteomics, as well as new research foci such as drug discovery, metabolomics, and ribosome sequencing analysis. We also observe a shift of research interest from developing tools for analyzing high-throughput data towards translational applications, as reflected in several papers that focus on bioinformatics analysis of large-scale data sets to test biological hypothesis regarding disease mechanisms. In the following, we briefly summarize the 13 selected papers.

## The science program for the ICIBM 2019 bioinformatics track

In the first paper by Ayed et al. [1] the authors developed a novel chemical representation method: Latent Target Interaction Profile (LTIP). Traditionally, chemical fingerprints represent a widely used feature to build machine learning models to predict response upon the chemical perturbation on a biological system in the field of computational drug discovery. However, chemical fingerprints that are derived from chemical structures ignore the biological context; thus, they suffer from several problems such as the activity cliff and curse of dimensionality. In comparison, LTIP embeds chemicals into a low dimensional continuous latent space that represents genome-scale chemical-target interactions, so that it can be used as a feature to build machine learning models. To evaluate this approach, the authors used drug sensitivity of cancer cell lines as a benchmark, and demonstrated that the LTIP robustly outperforms chemical fingerprints regardless of machine learning algorithms. Importantly, LTIP may also play complementary role with the chemical fingerprints, so that it can be combined with other fingerprints to further improve the performance of bioactivity prediction. The authors concluded that LTIP in particular and multi-scale modeling in general has a strong potential in predictive modeling of chemical modulation of biological activities.

In the next paper by Feng et al. [2] the authors developed MIRIA, a webserver for statistical, visual and meta-analysis of RNA editing data in mammals. RNA editing is defined as a critical post-transcriptional regulatory RNA-processing event (excluding RNA splicing) that generates an RNA transcript with a primary nucleotide sequence different from its gene. In mammals, the most common form of RNA editing is A-to-I RNA editing, and this process leads to an A-to-G reading of the cDNA molecule. To facilitate the identification of RNA editing sites as well as downstream analysis, the authors developed MIRIA as a user-friendly webserver that integrates statistics and visualization techniques to facilitate the comprehensive analysis of RNA editing site data identified by the analysis pipelines and software packages. MIRIA is unique in that it provides several analytical functions, including RNA editing type statistics, genomic feature annotations, editing level statistics, genome-wide distribution of RNA editing sites, tissue-specific analysis and conservation analysis. To demonstrate the effectiveness of the method, they collected high-throughput RNA sequencing (RNA-seq) data from eight tissues across seven species as the experimental data for MIRIA and constructed an example result page. In summary, MIRIA provides both visualization and analysis of mammal RNA editing data for experimental biologists who are interested in revealing the functions of RNA editing sites.

The next paper by Zhang et al. [3] describes M3S (Multi-Modal Model Selection), a comprehensive model selection for multi-modal single-cell RNA sequencing data. M3S is an R package for gene-wise selection of the most proper multi-modality statistical model and downstream analysis, useful in a single-cell or large-scale bulk tissue transcriptomic data. Several unique features of M3S are summarized here. First, it performs gene-wise selection of the most parsimonious model among 11 most commonly utilized ones that can best fit the expression distribution of the gene. Second, it carries out parameter estimation of a selected model. Third, it performs differential gene expression test based on multi-modality model. They benchmarked the M3S package on simulated data sets and four real single cell RNA-seq data sets, and showed that M3S has high specificity and can effectively identify the outlier features. Furthermore, they applied the M3S.test function to identify differentially expressed genes associated with pre-defined sample classes in the T cell scRNA-seq data set, and showed its effectiveness by comparing to other commonly used differential gene expression analysis methods for scRNA-seq. In summary, the M3S package may facilitate the downstream analysis of single-cell or large-scale bulk tissue transcriptomic data.

The next paper by Cui et al. [4] described a method called DeepShape for estimating isoform-level ribosome abundance and distribution with ribosome sequencing (ribo-seq) data. Ribo-seq is an important approach to obtain ribosome distributions by sequencing “ribosome-protected fragments” (RPFs). Computational analysis involves mapping sequencing reads into transcripts and obtaining the distributions of ribosomes along the whole transcriptome. Existing methods either discard the multiple mapped-reads, allocate them randomly, or assign them proportionally according to transcript abundance estimated from RNA-seq data. In comparison, DeepShape is an RNA-seq free computational method to estimate ribosome abundance of isoforms, and it simultaneously computes their ribosome profiles using a deep learning model. Simulation studies and real data analysis demonstrated that DeepShape can provide more accurate estimations on both ribosome abundance and profiles when compared to state-of-the-art methods. Therefore, DeepShape can serve as a powerful tool for Ribo-seq data analysis.

The next paper by Wu et al. [5] presents SigUNet for signal peptide recognition based on semantic segmentation. Proteins with signal peptides can enter the secretory pathway and then be transported to appropriate organelles where the proteins are able to conduct their functions. Signal peptides work as a permission gateway that transport proteins into endoplasmic reticulum. Recognition of signal peptides is an important first step to understand the active locations and functions of proteins. Many computational methods including deep learning methods have been proposed to help signal peptide recognition. However, most of existing neural network models for signal peptide recognition are relatively simple without leveraging the developments of other fields. This work proposes a convolutional neural network architecture without fully connected layers, which is an important architecture improvement in computer vision. The experiments results showed that the proposed model outperforms conventional neural networks on eukaryotic signal peptides, but the advantage is not observed on bacterial signal peptides because of the small data size. In summary, this study developed an accurate signal peptide recognizer, demonstrated the potential of leveraging advanced networks from other fields, and made important modifications while using advanced networks on signal peptide recognition.

The next paper by Liu et al. [6] described a unified short tandem repeats (STRs) profiling system across multiple species with whole genome sequencing data. STRs, also referred to as simple sequence repeats (SSRs) or microsatellite loci, are DNA fragments made up of tandem repeats of 1–6 bp sequence units. STRs are ubiquitous in eukaryotic genomes and are highly polymorphic. A variety of STRs profiling systems have been developed for species including human, dog, cat, cattle, etc. To maintain these systems simultaneously can be costly. These mammals share many high similar regions along their genomes, so it is possible to develop a unified STR profiling system. In this study, the authors proposed and developed a unified set of STR loci that could be simultaneously applied to multiple species. They presented an algorithm which selected a subset of loci by incorporating the optimized combined power of discrimination. Their results showed that the unified set of loci have high combined power of discrimination for both individual species and the mixed population, indicating that the identified set of STR loci could be applied to multiple species. In summary, through bioinformatics analysis of whole-genome sequencing data, the newly developed unified STR profiling system can be applied to the individual identification or paternal test of each of the ten common species: *Sus scrofa* (pig), *Bostaurus* (cattle), *Capra hircus* (goat), *Equus caballus* (horse), *Canis lupus familiaris* (dog), *Felis catus* (cat), *Ovis aries* (sheep), *Oryctolagus cuniculus* (rabbit), *Bos grunniens* (yak), and *Homo sapiens* (human).

The next paper by Abrams et al. [7] described a bioinformatics protocol to evaluate RNA sequencing normalization methods. For RNA-Seq data analysis, normalization methods are typically used for reducing the non-biologically derived variability inherent in transcriptomic measurements. However, the comparative efficacy of the various normalization techniques has not been tested in a standardized manner. In the study, the authors proposed tests that evaluate several normalization techniques on a large-scale standard data set. They concluded that transcripts per million (TPM) was the best performing normalization method based on its preservation of biological signal as compared to the other methods tested. With the proposed bioinformatics schema presented in the paper, researchers can evaluate their own or future normalization methods to further improve the field of RNA-Seq data analysis.

The next paper by Yu et al. [8] proposed a fully moderated t-statistic in linear modeling of mixed effects for differential expression analysis. Typical gene expression profiling experiments with few replicates lead to great variability in the estimates of gene variances, and several moderated t-test methods based on linear models with mixed effects have been developed to reduce this variability and to increase power of tests of differential expression. However, they are inadequate for designs with complex correlation structures, therefore application of moderation methods to linear models with mixed effects are needed for differential expression analysis. In the study, the authors implemented a fully moderated t-statistic method for linear models with mixed effects, where both residual variances and variance estimates of random effects are smoothed under a hierarchical Bayes framework. They showed that the proposed method can control the expected number of false positives at the nominal level, while two other currently used moderation methods fail. In summary, the proposed method is able to improve power by moderation and control the expected number of false positives properly at the nominal level.

The next paper by Klein et al. [9] performed bioinformatics analysis to study the association between amyotrophic lateral sclerosis (ALS) and retroviruses. ALS is a progressive neurodegenerative disorder affecting motor neurons of the brain and spinal cord. To date, approximately 10–15% of ALS cases have been found to have a genetic basis, and causal mutations have been identified in ~ 70% of familial cases and ~ 10% of sporadic cases. Emerging evidence suggests retroviruses play a role in the pathophysiology of ALS. Specifically, activation of ancient viral genes embedded in the human genome is theorized to lead to motor neuron degeneration. To test this hypothesis, the authors explored whether connections exist between ALS and retroviruses through protein interaction networks (PIN) and pathway analysis, and consider the potential roles in drug target discovery. Topological and statistical analysis of the ALS-PIN and retrovirus-PIN identified a shared, essential protein network and a core cluster with significant connections with both networks. Pathway enrichment analysis showed that this core cluster is associated with the glucocorticoid receptor singling and neuroinflammation signaling pathways. The same methodology was applied to the West Nile and Polio virus, which demonstrated trivial connectivity with ALS, supporting the unique connection between ALS and retroviruses. In summary, bioinformatics analysis provides evidence to support pathological links between ALS and retroviral activation, and suggested the important roles of the neuroinflammation and apoptotic regulation pathways. The continuation and further analysis of large-scale genome studies may prove useful in exploring genes that are important in retroviral activation and ALS, which may help discover new drug targets.

The next paper by Shah et al. [10] presented BayesMetab, a bioinformatics method for the treatment of missing values in metabolomic studies using a Bayesian modeling approach. Missing values (MVs) are pervasive, yet the treatment of MVs can have a substantial impact on downstream statistical analyses of metabolomics data. The MVs problem in metabolomics can arise because the metabolite is not biologically present in the sample, or is present in the sample but at a concentration below the lower limit of detection (LOD), or is present in the sample but undetected due to technical issues related to sample pre-processing steps. The former is considered missing not at random (MNAR) while the latter is an example of missing at random (MAR). Typically, such MVs are substituted by a minimum value, which may lead to severely biased results in downstream analyses. Here, the authors developed a Bayesian model that systematically accounts for missing values based on a Markov chain Monte Carlo (MCMC) algorithm that incorporates data augmentation by allowing MVs to be due to either truncation below the LOD or other technical reasons unrelated to its abundance. Their simulation results indicate that the proposed Bayesian method outperformed other imputation algorithms when there is a mixture of missingness due to MAR and MNAR. Further, their approach was competitive with other methods tailored specifically to MNAR in situations where missing data were completely MNAR. Their method was also tested on a real metabolomics dataset from a mouse myocardial infarction, and it revealed several statistically significant metabolites not previously identified but with direct biological relevance to the study. In summary, these findings demonstrate that the proposed Bayesian method has improved performance in imputing the missing values and performing statistical inference compared to other current methods when missing values are due to a mixture of MNAR and MAR.

The next paper by Gadepalli et al. [11] described BISR-RNAseq, an efficient and scalable RNA-Seq analysis workflow with interactive report generation. The BISR-RNAseq workflow implements several open-source software tools that can be run on a high performance computing environment. The workflow allows for the analysis (alignment, quality control, gene-wise counts generation) of raw RNA-Seq data and seamless integration of quality analysis and differential expression results into a configurable R shiny web application. Scripts are set up to run in a high performance computing (HPC) environment that utilizes portable batch system (PBS) for job scheduling and could be easily modified for other schedulers such as slurm. To further evaluate the effectiveness of the workflow, the authors applied the pipeline to a few publicly available RNA-seq datasets downloaded from Gene Expression Omnibus (GEO) and demonstrated how the workflow could facilitate users to perform RNA-seq data analysis in a user-friendly interface.

The next paper by Church et al. [12] tried to understand gene expression heterogeneity by investigating skewness in large patient cohorts. Skewness is a statistical measure that captures the degree of asymmetry in the distribution of any dataset. This study applied a new metric based on skewness to identify regulators or genes that have outlier expression in large patient cohorts. The authors investigated whether specific patterns of skewed expression were related to the enrichment of biological pathways or genomic properties such as DNA methylation status. The authors used several publicly available datasets that were generated using both RNA-seq and microarray technology platforms. When comparing the shift in expression skewness between cancer and control datasets, they observed an enrichment of pathways related to immune function that reflect increases towards positive skewness in the cancer relative to control datasets. Significant correlation was also detected between expression skewness and differential DNA methylation occurring in the promotor regions for four TCGA cancer cohorts. In summary, these results indicate that expression skewness can reveal new insights into transcription based on outlier and asymmetrical behavior in large patient cohorts.

In the last paper, Eicher et al. [13] performed a comparison of analysis methods with the case study of the DREAM Proteogenomics Challenge data. Proteomic measurements provide insights into gene expression regulations and mechanisms underlying altered phenotypes, while the integration of proteome and transcriptome data can validate gene signatures associated with a phenotype. However, proteomic data is not as abundant as genomic data, and it is thus beneficial to use genomic features to predict protein abundances when matching proteomic samples or measurements within samples are lacking. In the current study, the authors evaluated and compared three data-driven models for prediction of proteomic data from mRNA in breast and ovarian cancers using the 2017 DREAM Proteogenomics Challenge datasets. Their results showed that Bayesian network and random forest approaches could predict protein abundance levels with median ground truth (predicted correlation values were between 0.29 and 0.55). Logic-based predictors were not as accurate overall, but performed well for a subset of proteins across multiple cross-validations. In summary, they benchmarked several machine learning approaches for predicting proteomic data from gene expression data, and discussed the challenges and potential solutions in state-of-the-art proteogenomic analyses.

## References

[1] Ayed M, Lim H, Xie L. Biological representation of chemicals using latent target interaction profile. BMC Bioinformatics. 2019;20(s24). 10.1186/s12859-019-3241-3. 10.1186/s12859-019-3241-3 PMC6924142 31861982

[2] Feng X, Wang Z, Li H, Li S. MIRIA: a webserver for statistical, visual and meta-analysis of RNA editing data in mammals. BMC Bioinformatics. 2019;20(s24). 10.1186/s12859-019-3242-2. 10.1186/s12859-019-3242-2 PMC6923819 31861975

[3] Zhang Y, Wan C, Wang P, Chang W, Huo Y, Chen J, Ma Q, Cao S, Zhang C. M3S: A comprehensive model selection for multi-modal single-cell RNA sequencing data. BMC Bioinformatics. 2019;20(s24). 10.1186/s12859-019-3243-1. 10.1186/s12859-019-3243-1 PMC6923906 31861972

[4] Cui H, Hu H, Zeng J, Chen T. DeepShape: estimating isoform-level ribosome abundance and distribution with Ribo-seq data. BMC Bioinformatics. 2019;20(s24). 10.1186/s12859-019-3244-0. 10.1186/s12859-019-3244-0 PMC6923924 31861979

[5] Wu J, Liu Y, Chang T. SigUNet: signal peptide recognition based on semantic segmentation. BMC Bioinformatics. 2019;20(s24). 10.1186/s12859-019-3245-z. 10.1186/s12859-019-3245-z PMC6923836 31861981

[6] Liu Y, Xu J, Li S. A unified STR profiling system across multiple species with whole genome sequencing data. BMC Bioinformatics. 2019;20(s24). 10.1186/s12859-019-3246-y. 10.1186/s12859-019-3246-y PMC6923897 31861983

[7] Abrams Z, Johnson T, Huang K. Philip Payne, Kevin Coombes. A protocol to evaluate RNA sequencing normalization methods. BMC Bioinformatics. 2019;20(s24). 10.1186/s12859-019-3247-x. 10.1186/s12859-019-3247-x PMC6923842 31861985

[8] Yu L, Zhang J, Brock G, Fernandez S. Fully moderated T-statistic in linear modeling of mixed effects for differential expression analysis. BMC Bioinformatics. 2019;20(s24). 10.1186/s12859-019-3248-9. 10.1186/s12859-019-3248-9 PMC6923909 31861977

[9] Klein J, Sun Z, Staff N. Association between ALS and retroviruses: evidence from bioinformatics analysis. BMC Bioinformatics. 2019;20(s24). 10.1186/s12859-019-3249-8. 10.1186/s12859-019-3249-8 PMC6923964 31861978

[10] Shah J, Brock G, Gaskins J. BayesMetab: treatment of missing values in Metabolomic studies using a Bayesian modeling approach. BMC Bioinformatics. 2019;20(s24). 10.1186/s12859-019-3250-2. 10.1186/s12859-019-3250-2 PMC6923847 31861984

[11] Gadepalli VS, Ozer HG, Yilmaz AS, Pietrzak M, Webb A. BISR-RNAseq: an efficient and scalable RNAseq analysis workflow with interactive report generation. BMC Bioinformatics. 2019;20(s24). 10.1186/s12859-019-3251-1. 10.1186/s12859-019-3251-1 PMC6923898 31861980

[12] Church B, Williams H, Mar J. Investigating Skewness to understand gene expression heterogeneity in large patient cohorts. BMC Bioinformatics. 2019;20(s24). 10.1186/s12859-019-3252-0. 10.1186/s12859-019-3252-0 PMC6923883 31861976

[13] Eicher T, Patt A, Kautto E, Machiraju R, Mathe E, Zhang Y. Challenges in proteogenomics: a comparison of analysis methods with the case study of the DREAM Proteogenomics sub-challenge. BMC Bioinformatics. 2019;20(s24). 10.1186/s12859-019-3253-z. 10.1186/s12859-019-3253-z PMC6923881 31861998

