# Correction to: The International Conference on Intelligent Biology and Medicine (ICIBM) 2019: bioinformatics methods and applications for human diseases

**DOI:** 10.1186/s12859-020-3487-9

**Published:** 2020-04-16

**Authors:** Zhongming Zhao, Yulin Dai, Chi Zhang, Ewy Mathé, Lai Wei, Kai Wang

**Affiliations:** 1 Center for Precision Health, School of Biomedical Informatics, The University of Texas Health Science Center at Houston, Houston, TX 77030 USA; 2 Department of Medical and Molecular Genetics, Indiana University School of Medicine, Indianapolis, IN 46202 USA; 3 Department of Biomedical Informatics, The Ohio State University College of Medicine, Columbus, OH 43214 USA; 4 Raymond G. Perelman Center for Cellular and Molecular Therapeutics, Children’s Hospital of Philadelphia, Philadelphia, PA 19104 USA; 5 Department of Pathology and Laboratory Medicine, University of Pennsylvania, Philadelphia, PA 19104 USA

**Correction**

After publication of this supplement article [1], it is requested the grant ID in the Funding section should be corrected from NSF grant IIS-7811367 to NSF grant IIS-1902617.

Therefore, the correct ‘Funding’ section in this article should read:

We thank the National Science Foundation (NSF grant IIS-1902617) for the financial support of ICIBM 2019. This article has not received sponsorship for publication.
